# Supplementary material for: Preventive Effect of the Total Polyphenols from Nymphaea candida on Sepsis-Induced Acute Lung Injury in Mice via Gut Microbiota and NLRP3, TLR-4/NF-κB Pathway
Source: Int J Mol Sci. 2024 Apr 12;25(8):4276. doi: 10.3390/ijms25084276 (PMC11050158; doi:10.3390/ijms25084276)
Supplement: Supplementary file 1 [file ijms-25-04276-s001.zip › ijms-2930536-supplementary.pdf]

Supplementary Materials

This supplementary materials file contains the following contents:

Supplementary Tables	Table S1 to S11
Supplementary Figures	Figures S1 to S4

Table S1. Effect of NCTP on lung tissues MPO levels in LPS-induced ALI mice

Group	MPO(pg/mL)
Control	320.94±33.19
Model	540.52±84.76##
NCTP, 50mg/kg	537.01±91.14
NCTP, 100mg/kg	451.15±23.05*
NCTP, 200mg/kg	400.35±59.82**
DEX, 3 mg/kg	346.38±26.35**

Data are presented as mean±SD., *n*=6. ##*p*<0.01 versus control group, **p*<0.05 and ***p*<0.01 versus model group.

Table S2. Effect of NCTP on BALF total proten, LDH contents in ALI mice

Group	LDH(U/L)	BCA(mg/mL)
Control	3.93±0.58	0.058±0.03
Model	17.38±2.12##	0.229±0.08#
NCTP, 50mg/kg	6.92±2.06**	0.105±0.02
NCTP, 100mg/kg	6.75±1.46**	0.094±0.05
NCTP, 200mg/kg	5.77±1.59**	0.066±0.03*
DEX, 3 mg/kg	5.50±1.73**	0.065±0.04*

Data are presented as mean±SD., *n*=6. #*p*<0.05 and ##*p*<0.01 versus control group, **p*<0.05 and ***p*<0.01 versus model group.

Table S3. Effects of inflammatory factors in blood in mice with ALI.

Group	WBC(10^9/L)	NEU(10^9/L)	MON(10^9/L)	BAS(10^9/L)	EOS(10^9/L)	LYM(10^9/L)
Control	3.38±0.68	0.97±0.07	0.17±0.02	0.023±0.01	0.08±0.02	2.81±0.70
Model	5.47±0.58##	3.29±0.32##	0.26±0.03##	0.052±0.01##	0.21±0.05#	1.12±0.13#
NCTP, 50mg/kg	3.80±0.13*	2.54±0.17*	0.22±0.02**	0.027±0.01**	0.15±0.03	1.28±0.27
NCTP, 100mg/kg	3.76±0.22**	2.16±0.13**	0.20±0.03**	0.022±0.01**	0.11±0.02*	1.62±0.93
NCTP, 200mg/kg	3.38±0.37**	2.10±0.25**	0.17±0.03**	0.018±0.01**	0.10±0.02*	1.70±0.25*
DEX, 3 mg/kg	3.42±0.76**	1.84±0.50**	0.20±0.03**	0.027±0.01*	0.10±0.02*	1.89±0.87

Data are presented as mean±SD., *n*=6. #*p*<0.05 and ##*p*<0.01 versus control group, **p*<0.05 and ***p*<0.01 versus model group.

Table S4. Effects of inflammatory factors in blood in mice with ALI

Group	TNF-α(pg/mL)	IL-1β(pg/mL)	IL-6(pg/mL)	LPS(EU/L)
Control	229.16±41.40	14.19±2.96	27.12±4.60	5.66±0.74
Model	461.10±41.22##	42.84±6.86##	63.71±8.46##	12.68±2.06##
NCTP, 50mg/kg	360.21±37.02**	32.70±6.10**	43.84±5.20**	6.51±0.30*
NCTP, 100mg/kg	344.13±50.69**	27.42±4.33**	37.49±6.10**	5.76±0.30**
NCTP, 200mg/kg	339.03±76.31**	22.66±3.32**	33.05±5.22**	5.53±0.40**
DEX, 3 mg/kg	323.69±69.11**	20.62±4.46**	24.41±2.83**	5.87±0.56**

Data are presented as mean±SD., *n*=6. ##*p*<0.01 versus control group, **p*<0.05 and ***p*<0.01 versus model group.

Table S5. Effects of NCTP on proteins expression of TLR-4/NF-κB p65 in the lung tissues of mice

Group	TLR 4	MyD 88	TRAF6	IKK	p-Iκ B	p-NF-κB p65
Control	0.35±0.06	0.47±0.11	0.42±0.11	0.49±0.14	0.51±0.05	0.65±0.13
Model	1.07±0.16##	1.07±0.08##	0.95±0.11##	1.12±0.05##	1.37±0.17##	1.18±0.06##
NCTP, 50mg/kg	0.96±0.13	0.83±0.04**	0.81±0.23	0.79±0.19**	0.87±0.19**	1.12±0.28
NCTP, 100mg/kg	0.92±0.16	0.73±0.08**	0.81±0.22	0.67±0.16**	0.78±0.04**	0.84±0.30
NCTP, 200mg/kg	0.73±0.25*	0.46±0.09**	0.45±0.15**	0.46±0.13**	0.61±0.28**	0.63±0.31*
DEX, 3 mg/kg	0.71±0.25*	0.44±0.18**	0.40±0.18**	0.54±0.28**	0.70±0.10**	0.75±0.01*

Data are presented as mean±SD., *n*=6. ##*p*<0.01 versus control group, **p*<0.05 and ***p*<0.01 versus model group.

Table S6. Effects of NCTP on proteins expression of NLRP3 in lung tissues of mice

Group	NLRP3	ASC	Caspase1
Control	0.34±0.05	0.25±0.09	0.40±0.26
Model	1.03±0.06##	0.91±0.12##	0.99±0.24##
NCTP, 50mg/kg	0.92±0.16	0.70±0.25	0.84±0.10
NCTP, 100mg/kg	0.72±0.03**	0.60±0.12*	0.87±0.16
NCTP, 200mg/kg	0.51±0.07**	0.43±0.21**	0.57±0.03*
DEX, 3 mg/kg	0.61±0.15**	0.28±0.14**	0.53±0.19**

Data are presented as mean±SD., *n*=6. ##*p*<0.01 versus control group, **p*<0.05 and ***p*<0.01 versus model group.

Table S7. Effect of NCTP on the levels of LBP in intestinal tissue

Group	LBP(pg/mL)
Control	3.16±0.84
Model	10.84±1.10##
NCTP, 50mg/kg	5.33±0.29**
NCTP, 100mg/kg	4.58±0.27**
NCTP, 200mg/kg	4.31±0.43**
DEX, 3 mg/kg	3.89±0.71**

Data are presented as mean±SD., *n*=6. ##*p*<0.01 versus control group, ***p*<0.01 versus model group.

Table 8. Effects of NCTP on ZO-1 and occludin protein expression in ALI induced by LPS.

Group	ZO-1	occludin

Control	0.21±0.01	0.21±0.01
Model	0.12±0.00##	0.14±0.00##
NCTP, 50mg/kg	0.15±0.00**	0.15±0.00**
NCTP, 100mg/kg	0.17±0.00**	0.17±0.00**
NCTP, 200mg/kg	0.18±0.00**	0.19±0.00**
DEX, 3 mg/kg	0.19±0.00**	0.19±0.01**

Data are presented as mean±SD., *n*=6. ##p<0.01 versus control group, **p<0.01 versus model group.

Table S9. Effects of NCTP on the gut microbiota dysbiosis of alpha diversity index

Group	Chao1	Simpson	Shannon	Observed_species
Control1	533.35	0.965214	6.85346	528.9
Control2	487.599	0.978034	7.08779	479.8
Control3	553.191	0.985501	7.24638	551
Model1	443.638	0.945964	6.25473	431.7
Model2	547.583	0.984731	7.26153	539.2
Model3	472.325	0.92114	5.67889	449.3
NCTP, 200mg/kg1	552.886	0.983559	7.09909	526.5
NCTP, 200mg/kg2	389.112	0.888746	5.13834	381.9
NCTP, 200mg/kg3	597.913	0.989211	7.5378	582.1

Table S10. The composition of gut microbiota of community bar plot analysis on phylum level

Group	Bacteroidetes	Firmicutes	Proteobacteria	Actinobacteria	Other
Control1	0.45051	0.40050	0.11959	0.02472	0.00468
Control2	0.61329	0.26322	0.08210	0.01521	0.02619
Control3	0.54347	0.36662	0.05738	0.01370	0.01883
Model1	0.51545	0.21035	0.25053	0.01801	0.00565
Model2	0.44250	0.22552	0.31425	0.01033	0.00740
Model3	0.41627	0.11459	0.40070	0.00679	0.06165
NCTP, 200mg/kg1	0.23052	0.53269	0.21080	0.01614	0.00984
NCTP, 200mg/kg2	0.41017	0.47890	0.07807	0.01708	0.01578
NCTP, 200mg/kg3	0.44510	0.42990	0.10853	0.00842	0.00805

Table S11. Effect of NCTP in the fecal contents levels of short-chain fatty acids

Group	Acetic acid	Propionic acid	Butyric acid	Isobutyric acid	Valeric acid	Isovaleric acid
Control1	780.0279	158.0120	147.1530	13.8854	21.5734	13.0666
Control2	615.1347	111.2760	117.3162	9.7719	12.7789	7.0137
Control3	963.8725	245.8098	79.1292	41.5373	30.7864	53.4256
Model1	125.3740	34.4466	14.9533	4.6361	4.1536	4.5953
Model2	172.6084	41.5371	19.0487	5.3474	4.6247	6.6614
Model3	137.9557	39.7339	13.8800	5.1333	4.9555	6.4373
NCTP, 200mg/kg1	732.9527	194.1088	79.1453	22.1424	22.3399	28.8777
NCTP, 200mg/kg2	575.5987	144.6668	51.8569	20.2049	12.5103	26.1495
NCTP, 200mg/kg3	644.0623	161.5644	67.8614	19.6699	15.9855	22.9044

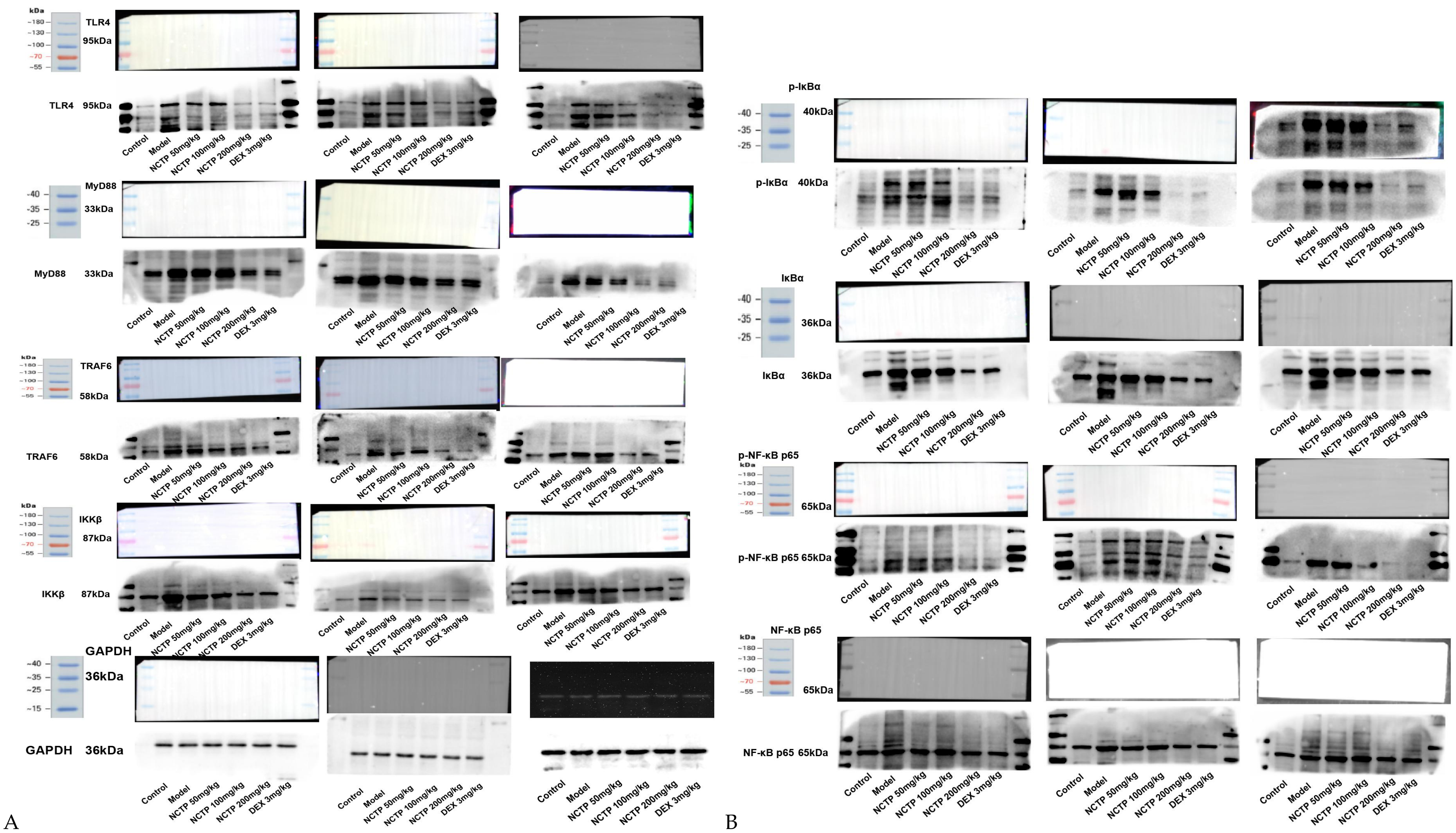

Figure S1. Effects of NCTP on proteins expression of lung tissues.(A) TLR4, MyD88, TRAF6, and IKKβ. (B) The phosphorylation of NF-κB p65 and IκB-α in ALI mic**e.**

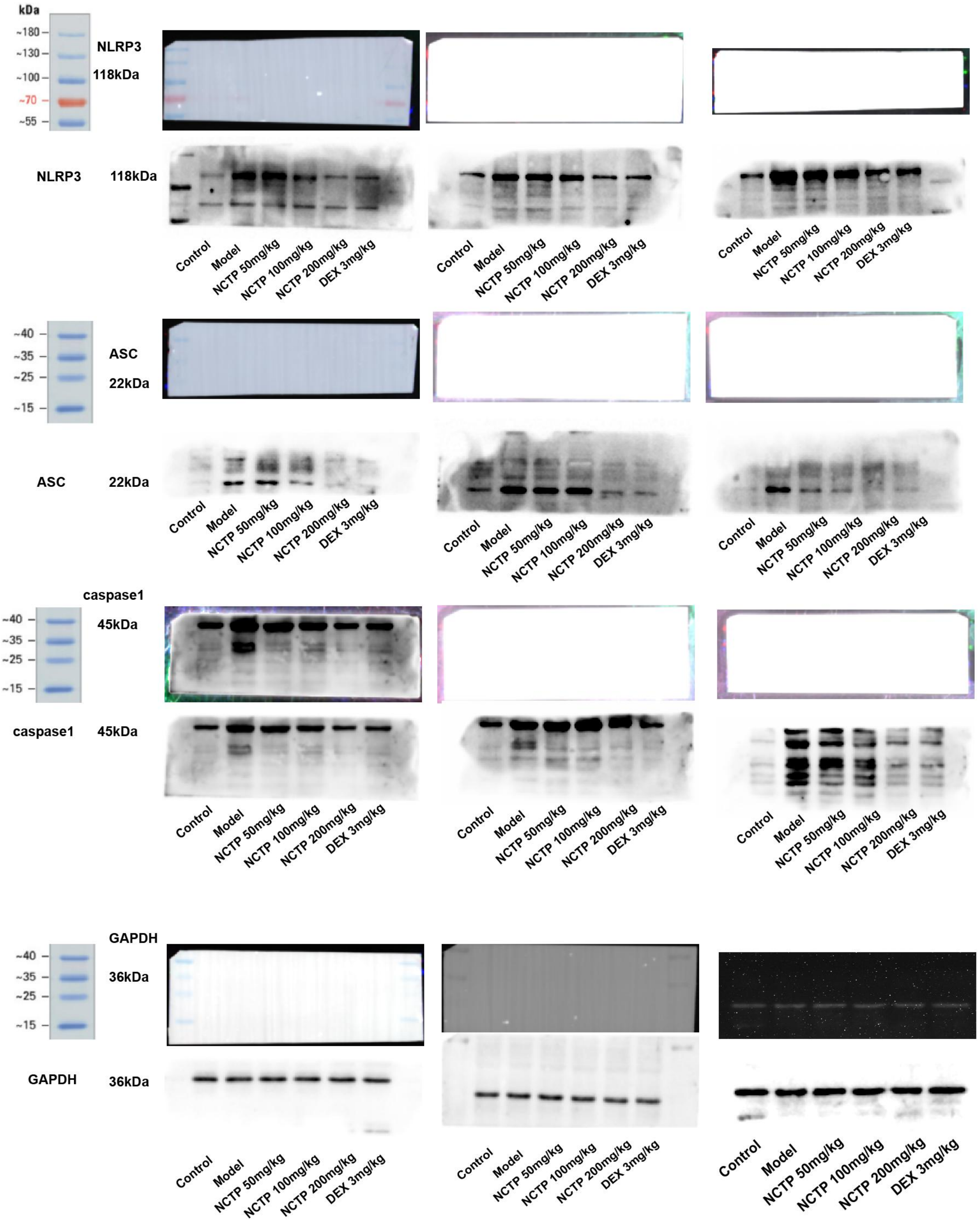

Figure S2. Effects of NCTP on proteins expression of NLRP3 in lung tissues of mice

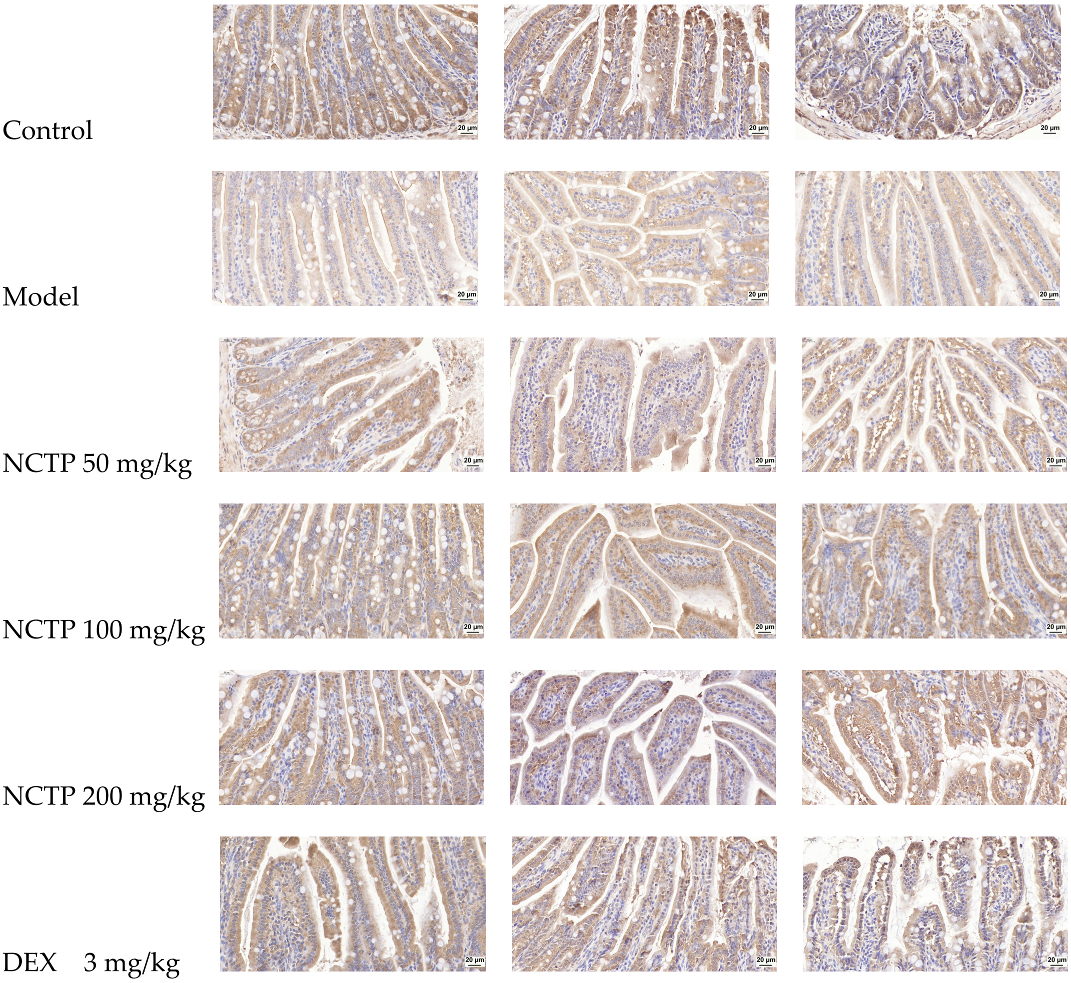

Figure S3. Effects of NCTP on ZO-1 protein expression in ALI mice.

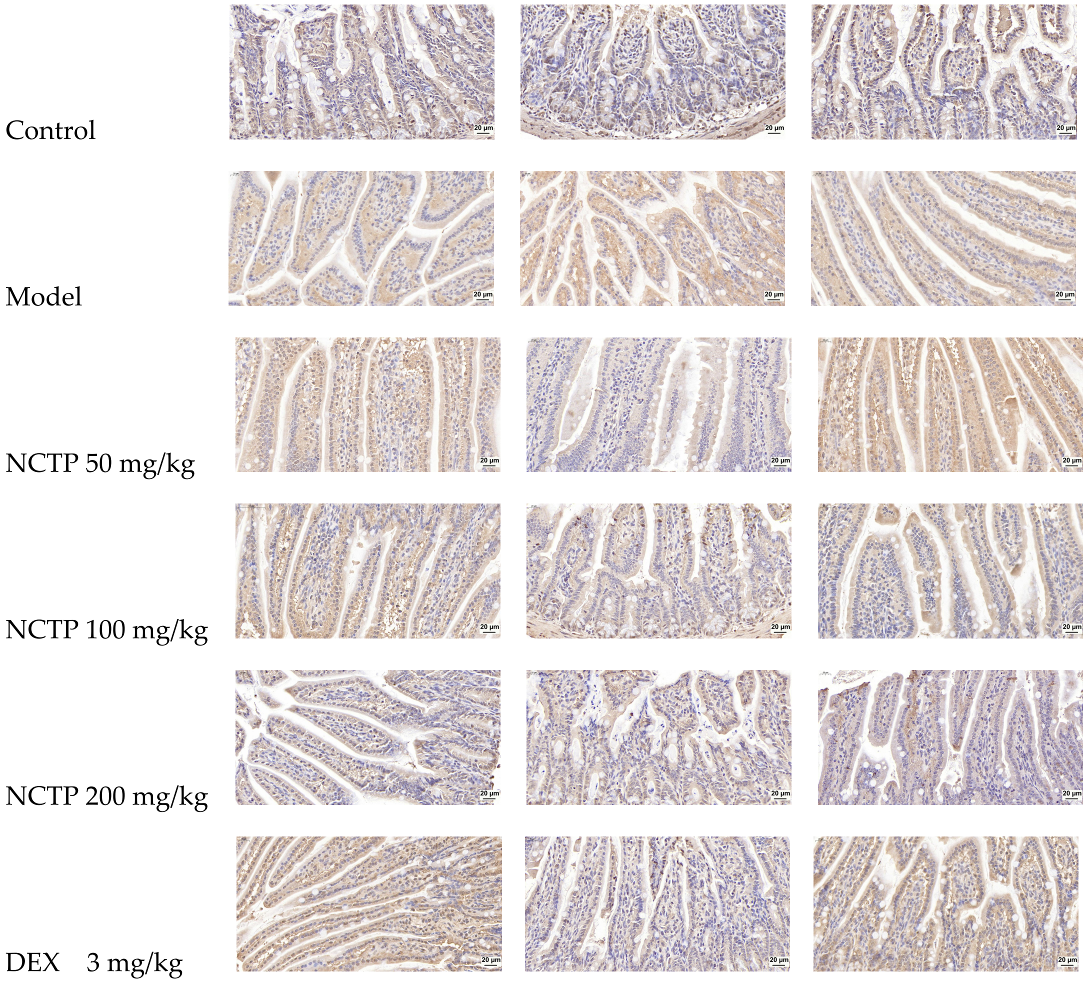

Figure S4. Effects of NCTP on occludin protein expression in ALI mice.
